# Effect of B cell depletion using peptide tetramers in collagen-induced arthritis

**DOI:** 10.1186/ar3634

**Published:** 2012-02-09

**Authors:** Kazuya Michishita, Kimito Kawahata, Takeyuki Kanzaki, Lisa Akahira, Toshiki Eri, Kazuhiko Yamamoto

**Affiliations:** 1 Department of Allergy and Rheumatology, Graduate School of Medicine, the University of Tokyo, Tokyo, Japan

## Background

B cell depletion therapy is effective in the treatment of various autoimmune diseases. However, this therapy is shown to be associated with increased risk of adverse effects such as opportunistic infections. Therefore, in this study, we developed and analyzed the selective depletion therapy of pathogenic B cells using peptide tetramers in collagen-induced arthritis model.

## Methods

Since the antigenic targets of pathogenic antibodies are identified in collagen-induced arthritis (CIA) model, we developed toxin-conjugated peptide tetramers, which contained pathogenic epitope of mouse type II Collagen (CII). The male DBA/1J mice were immunized with bovine CII and injected with toxin-conjugated peptide tetramers on day 10 and day 20 after CIIimmunization.We analyzed the effect of toxin-conjugated peptide tetramers on the production of autoantibodies and clinical course of arthritis.

## Results

The incidence of arthritis was significantly lower (*P *< 0.05) in the tetramer-treated group than in the control group. The mean serum antibody levels for CII did not differ significantly, but there were significant differences in the anti-peptide antibodies over time.

## Conclusions

Peptide tetramer is effective in the selective depletion of antigen-specific B cells and decreased the incidence of arthritis in CIA model. Therefore, depletion of antigen-specific B cells using this strategy might be a new therapeutic intervention of autoimmune diseases.

